# A comparison of the demographics, injury patterns and outcome data for patients injured in motor vehicle collisions who are trapped compared to those patients who are not trapped

**DOI:** 10.1186/s13049-020-00818-6

**Published:** 2021-01-14

**Authors:** Tim Nutbeam, Rob Fenwick, Jason Smith, Omar Bouamra, Lee Wallis, Willem Stassen

**Affiliations:** 1 Emergency Department, University Hospitals Plymouth NHSTrust, Plymouth, UK; 2 Devon Air Ambulance Trust, Exeter, UK; 3 University Hospitals Birmingham, Birmingham, UK; 4 Academic Department of Military Emergency Medicine, Royal Centre for Defence Medicine, Birmingham, UK; 5 Trauma Audit Research Network, University of Manchester, Manchester, UK; 6 Division of Emergency Medicine, University of Cape Town, Cape Town, South Africa

**Keywords:** Extrication, Road traffic collision, Spinal injury, Cervical collars, Pre-hospital care

## Abstract

**Background:**

Motor vehicle collisions (MVCs) are a common cause of major trauma and death. Following an MVC, up to 40% of patients will be trapped in their vehicle. Extrication methods are focused on the prevention of secondary spinal injury through movement minimisation and mitigation. This approach is time consuming and patients may have time-critical injuries. The purpose of this study is to describe the outcomes and injuries of those trapped following an MVC: this will help guide meaningful patient-focused interventions and future extrication strategies.

**Methods:**

We undertook a retrospective database study using the Trauma Audit and Research Network database. Patients were included if they were admitted to an English hospital following an MVC from 2012 to 2018. Patients were excluded when their outcomes were not known or if they were secondary transfers.

**Results:**

This analysis identified 426,135 cases of which 63,625 patients were included: 6983 trapped and 56,642 not trapped. Trapped patients had a higher mortality (8.9% vs 5.0%, *p* < 0.001). Spinal cord injuries were rare (0.71% of all extrications) but frequently (50.1%) associated with other severe injuries. Spinal cord injuries were more common in patients who were trapped (*p* < 0.001).

Injury Severity Score (ISS) was higher in the trapped group 18 (IQR 10–29) vs 13 (IQR 9–22). Trapped patients had more deranged physiology with lower blood pressures, lower oxygen saturations and lower Glasgow Coma Scale, GCS (all *p* < 0.001). Trapped patients had more significant injuries of the head chest, abdomen and spine (all p < 0.001) and an increased rate of pelvic injures with significant blood loss, blood loss from other areas or tension pneumothorax (all *p* < 0.001).

**Conclusion:**

Trapped patients are more likely to die than those who are not trapped. The frequency of spinal cord injuries is low, accounting for < 0.7% of all patients extricated. Patients who are trapped are more likely to have time-critical injuries requiring intervention. Extrication takes time and when considering the frequency, type and severity of injuries reported here, the benefit of movement minimisation may be outweighed by the additional time taken. Improved extrication strategies should be developed which are evidence-based and allow for the expedient management of other life-threatening injuries.

## Background

Motor Vehicle Collisions (MVCs) are the second most common cause of major trauma in the United Kingdom (UK) [1]. Following an MVC, patients within the car prior to the incident occurring can be ejected from the car, leave the car with or without assistance, or may remain in the vehicle. Patients who remain within their vehicle and cannot leave without assistance are considered ‘trapped’.

When a patient is trapped in a vehicle, they are considered at higher risk of significant injury than patients who are not trapped. Prolonged entrapment and/or intrusion into the patient compartment is considered high risk for significant injury and therefore features as part of the risk stratification of commonly used major trauma decision-making tools [2–9]. Fire and Rescue Service (FRS) delivered extrication strategies have evolved based on the paradigm of movement mitigation to avoid exacerbation of potential spinal injury; such strategies can take a significant amount of time (median 30, IQR 24–38 min [10]). FRS teaching mandates that all casualties should be considered to have spinal trauma (and therefore subject to an extrication) until proven otherwise [11].

Patients who are trapped after an MVC may have other time-critical injuries which are not amenable to intervention whilst the patient remains trapped – furthermore, being trapped prolongs scene time with a subsequent delay in accessing definitive care, such as surgical haemostasis [12]. Currently there is a paucity of evidence regarding the rate and type of spinal injuries of those trapped following an MVC, furthermore, we do not have a good understanding of the type and rate of time-critical injuries within this group. Without this understanding extrication approaches cannot be contextualised or understood in terms of potential benefits and harms to our patients.

This study aims to compare the demographics, 30-day mortality, rate and type of spinal injuries and other time-critical injuries between patients trapped and not trapped following an MVC from a UK based national trauma registry. These data will be compared with nationally reported FRS data to understand the number of patients trapped who have major trauma compared to the total number of extrications performed.

## Methods

We undertook a retrospective database study using the Trauma Audit and Research Network (TARN) database. TARN is a UK trauma registry to which all Major Trauma Centres (MTCs) submit data in order to access patient specific tariffs. Since the inception of trauma networks in the UK in 2012, TARN moved from voluntary to mandatory submission of data from participating centres. Eligibility for inclusion on the TARN database includes trauma patients who are admitted to hospital for ≥72 h, are admitted to a critical care unit, who die in hospital or are transferred to another hospital for specialist care. Patients aged over 65-years with isolated closed fractures of the limbs and hip fractures are excluded from the TARN dataset. TARN includes data on mechanism of injury, which allows patients with certain categories of injuries (e.g. post MVC) to be identified and analysed. MVCs are the second most common cause of trauma recorded on the TARN database (after ground level falls).

TARN uses an outcome prediction model including known confounders of trauma outcomes such as age, gender, injury severity score (ISS), Glasgow Coma Scale (GCS) and Charlson comorbidity index as independent predictors [13]. This allows the calculation of a Probability of Survival for every patient. This is used to build up a Performance Indicator (Ws) which compares groups of patients or institutions. The Ws is used to compare the performance of trauma networks and major trauma centres. The Ws is a directly standardised excess survival rate derived from a difference between the observed and expected number of survivors per 100 patients. A positive value of Ws indicates that the institution has more survivors than predicted, and so its performance is above the standard in the prediction database. The Ws was used in the context of this study to compare outcomes between patients trapped and not trapped, compensating for the confounders listed above.

The TARN database was interrogated to identify major trauma patients who were admitted between January 2012 and December 2018. Patients were excluded whose outcomes were not known, who were admitted outside England, who were not admitted directly and who were not involved in MVCs. Remaining patients were divided into three groups: trapped patients, patients who were not trapped, and those where the status was not recorded. Patients where the entrapment data were not recorded were excluded from further analysis.

Simple descriptive analysis was used to define the characteristics of the trapped and non-trapped groups. The Odds Ratio (OR) was calculated for patient mortality with the Ws used to demonstrate any excess survival difference accounting for included confounders. Levene’s test was used to assess equality of variances and a two-tailed t-test to compare means and Mann-Whitney test for comparing medians. Chi square test was used for categorical variables. *P* values of less than 0.05 were considered significant. SPSS software was used for the analysis.

TARN data analyses are conducted using anonymised data which is governed by a code of practice approved by the Confidentiality Advisory Group who are appointed by the Health Research Authority. Additional individual ethical approval was not required for this analysis.

Routinely collected anonymised FRS data, which are reported by central government and available in the public domain, were interrogated to identify the total number of extrications performed in 2012–2018 [14]. Simple analysis was used to describe these numbers in context of the spinal and time-critical injury analysis performed.

## Results

**Fig. 1:**
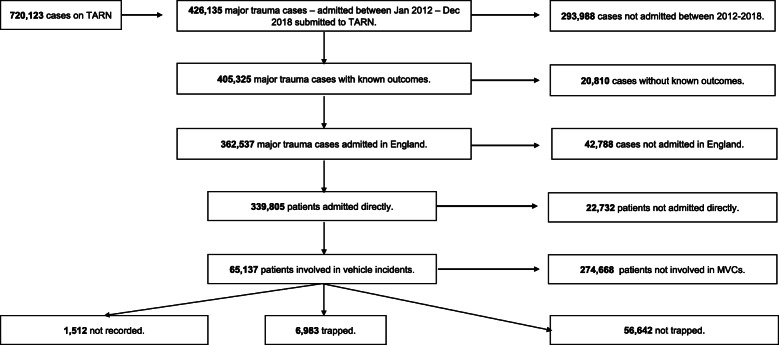
Strobe Diagram

During the study period, 426,135 major trauma cases were identified on the TARN database. Of these, 65,137 patients were admitted to hospital as a result of an MVC, and in 1512 the trapped status was not recorded (Fig. 1).

The characteristics of each group are summarized in Table 1. The median age (IQR) across all eligible patients was 42.4 (25.1–58.8) years and 73.7% were male. Of patients who survived to hospital, 3568 (5.4%) died within 30 days of initial injury. Across the groups, the mean pre-hospital systolic blood pressure was 131 mmHg, respiratory rate 19 breaths per minute, oxygen saturations 98% with a median GCS of 15.

**Table 1:** Demographics and Mortality by Trapped Status

	Trapped		Not trapped		Sig /*p* value
Number of patients (%)	6983	(11.0%)	56,642	(89.0%)	–
Male n (%)	4374	(62.6%)	42,656	(75.3%)	–
Mean Age years (STD DEV)	44.2	(21.3)	43.4	(21.3)	0.003
Median ISS (IQR)	18	(10–29)	13	(9–22)	< 0.001
Systolic Blood Pressure mmHg (STD DEV)	129	(31)	133	(27)	< 0.001
Respiratory Rate (STD DEV)	21	(7.9)	20	(6.8)	< 0.001
Oxygen Saturations % (STD DEV)	94.8%	(10.5)	96.3%	(7.5)	< 0.001
Median GCS (IQR)	15	(14–15)	15	(13–15)	< 0.001
Crude 90 day mortality n (%)	624	(8.9%)	2804	(5.0%)	< 0.001

Of the 63,625 patients with a trapped status recorded, 6983 (11.0%) were trapped, with 56,642 (89%) in the not trapped group. Statistically significant differences were found between the two groups across the parameters identified in Table 1: age (*p* = 0.003), systolic blood pressure (*p* < 0.001), respiratory rate (*p <* 0.001), oxygen saturations (*p <* 0.001) and GCS (*p <* 0.001). Being trapped was associated with a worse 30-day mortality outcome (trapped, 8.94%, not trapped 4.95%, OR 1.88 (95% CI 1.72–2.06). Corresponding adjusted excess survival score (Ws) for those that were not trapped was 0.56 (0.31–0.8), and for those that were trapped was − 0.79 (− 1.39 - -0.2). A negative score indicates that unexpected deaths occurred from what was predicted from the model.

As shown in Table 2, multiple spinal fractures, dens fractures, unstable spinal fractures and cord injuries all occurred more frequently in the trapped group (*p <* 0.001); this association did not reach statistical significance with compression fractures (*p* = 0.6).

**Table 2:** Time-critical and Spinal Injuries by Trapped Status

	Trapped		% of all Extrications^a^	Not trapped		Sig / *p* value:
Pelvic ring with blood loss > 20% n (%)	69	(1.0%)	0.16	370	(0.7%)	0.001
Blood loss > 20% n (%)	244	(3.5%)	0.56	1057	(1.9%)	< 0.001
Tension Pneumothorax n (%)	105	(1.5%)	0.24	472	(0.8%)	< 0.001
Multiple Spinal Fractures n (%)	942	(13.5%)	2.16	5003	(8.8%)	< 0.001
Spine Dens: Fracture n (%)	146	(2.1%)	0.33	586	(1.0%)	< 0.001
Spine: Compression Fracture n (%)	118	(1.7%)	0.27	1006	(1.8%)	0.606
Spine: Unstable Fracture n (%)	635	(9.1%)	1.46	3583	(6.3%)	< 0.001
Spine: Cord Injury n (%)	464	(6.6%)	0.71	2687	(4.7%)	< 0.001

^a^Percentage of all extrications performed during matched time period from FRS data

Of 464 trapped patients with a spinal cord injury, other significant injuries were present in 232 (50%) patients. The most commonly affected body area was thorax (48.6%) followed by head (24.3%), abdomen (9.7%) and pelvis (6.7%). Trapped patients with cord injuries rarely had concomitant time-critical injuries such as blood loss > 20% (1.7%), tension pneumothorax (1.5%) or pelvic injury with > 20% blood loss (0.6%).

The median ISS for all patients was 13 (IQR 9–24), and was significantly higher in the trapped group, 18 (IQR 10–29), when compared to the not trapped group, 13 (IQR 9–22, *p* < 0.001). As shown in Table 3, There was a statistically significant higher rate of severe (abbreviated injury scale (AIS) > = 3) injures to the head, chest, abdomen, pelvis, spine and limbs (all *p* < 0.001) in trapped patients compared to not trapped patients. The association was not present for those with a face AIS code of > = 3.

**Table 3:** Injury site by Trapped Status

Injury site^a^	Trapped		% of all Extrications^b^	Not trapped		Sig / *p* value:
Head AIS > = 3, n (%)	1742	(25.0%)	3.99	13,060	(23.1%)	< 0.001
Face AIS > = 3, n (%)	48	(0.7%)	0.11	307	(0.5%)	0.124
Chest AIS > = 3, n (%)	3699	(53.0%)	8.48	19,624	(34.7%)	< 0.001
Abdo AIS > = 3, n (%)	858	(12.3%)	1.97	4299	(7.6%)	< 0.001
Pelvis AIS > = 3, n (%)	738	(10.6%)	1.69	3487	(6.2%)	< 0.001
Spine AIS > = 3, n (%)	795	(11.4%)	1.82	4208	(7.4%)	< 0.001
Limb AIS > = 3, n (%)	2275	(32.6%)	5.21	16,668	(29.4%)	< 0.001

^a^Injuries are not mutually exclusive; patients may have more than one qualifying injury

^b^Percentage of all extrications performed during matched time period from FRS data that had these injuries

Trapped patients had a statistically significant higher frequency of pelvic ring injuries with blood loss > 20% (*p* < 0.001), other blood loss > 20% (*p <* 0.001) and tension pneumothorax (*p <* 0.001), though the rates of all three of these injuries were low in terms of total TARN patients and rare (all < 0.25%) when considering all the extrications reported in the UK FRS routinely reported data [14].

Table 4 shows that trapped patients more frequently underwent intubation, intercostal drain insertion, received tranexamic acid and blood product resuscitation than their non-trapped counterparts (*p* < 0.001).

**Table 4:** Time-critical Interventions by Trapped Status

	Trapped		Not trapped		Sig. /p
Intubation n (%)	1547	%)	6998	(12.4%)	< 0.001
Intercostal drain n (%)	656	(9.4%)	3099	(5.5%)	< 0.001
Administration of TXA n (%)	2871	(41.1%)	10,395	(18.4%)	< 0.001
Blood transfusion n (%)	1104	(15.8%)	3421	(6.0%)	< 0.001

## Discussion

This study has compared the demographics, 30-day mortality, rate and type of spinal injuries and other time-critical injuries between patients trapped and not trapped following an MVC from a UK based national trauma registry.

### Is being trapped associated with an increased mortality?

This study demonstrates a significantly higher mortality in the trapped population. This difference in mortality between the groups remains when known confounders considered in the Ws statistic are accounted for. Our results likely underestimate the effect of entrapment on mortality as patients who died on scene were not included in our analyses.

### Are spinal injuries common in patients who are trapped?

In high-income countries, patients who are trapped are extricated primarily by the FRS. The principles of extrication have developed without significant medical input [15] and they are based around movement minimization – specifically movement of the spine. Current FRS guidance suggests that even small movements are intolerable and all patients who have undergone trauma should be considered to have a spinal injury until proven otherwise [11]. This guidance accepts that other life-threatening injuries may be present, but the focus in extrication practice remains on the minimization of spinal movement.

Spinal injuries were infrequent in this study population, with trapped patients with a spinal cord injury representing just 0.71% (or one in 141) of all extrications performed. For the very small proportion of patients whom extrication techniques are targeted towards there is a very large number of patients with no or minor injuries (patients not on the TARN registry) whom as a result of application of movement minimization techniques consume significant resources. In addition, there is a large number of severely injured patients who have non-spinal or spinal and additional injuries who extrication approaches are not optimised for.

### Do patients with spinal injuries have other injuries which may dictate extrication needs?

In the context of prevention of secondary spinal injury, those patients who may benefit from movement minimization are those who have both a spinal cord injury and do not have other time-critical injuries that may take precedence when planning an extrication. This is a rare patient group; just 232 patients over the 6 years that this study covers, or 0.5% of the 43,633 total extrications (as recorded on the FRS database) that occurred. As isolated cord injury represents a small proportion of those who are trapped in their vehicles with injuries, extrication principles should therefore be reconsidered with a wider appreciation of the mortality and morbidity associated with other common injuries and injury patterns e.g. blood loss and tension pneumothorax. Within our data, for example, a trapped patient is five times more likely to have a chest AIS of 3+ than a spine AIS of the same severity (Table 3).

The findings of increased number and severity of injuries in those who are trapped are consistent with previous evidence. Palanca et al performed univariate and multivariate analysis on 621 patients involved in road traffic collisions presenting to a single centre. Two hundred and fifty-three patients had major injury defined as ISS > 15 [5]. They identified the need for extrication as an independent risk factor for severe injury (*p* < 0.0001; OR 2.9 (1.9–4.5)). In another large prospective study of 2363 patients, Lerner et al examined numerous pre-hospital factors associated with motor vehicle collisions [7]. They found that prolonged extrication (> 20 min) predicted MTC need with a sensitivity of 11% and a specificity of 98%, likelihood ratio 3.6 (2.2–5.9).

### Injuries in context of intervention when the patient remains trapped

A large number of patients in our study required life-saving interventions (OR (95% CI), such as intubation (2.02 (1.90–2.15)), decompression of a tension pneumothorax (1.79 (1.64–1.96), or blood product transfusion (2.92 (2.72–3.14), and trapped patients were more likely to require these interventions than their not trapped counterparts (*p* < 0.001). It is challenging to deliver these interventions safely and effectively to a patient that is trapped, due to the working environment, space constraints and inability to do a detailed physical examination. It has been suggested that rapid extrication, minimising the time the patient is trapped, may offer significant benefits. Kaiser et al reinforce this need in their report on 446 traumatically injured patients where they performed a regression analysis to predict the need for urgent surgery [16]. They identified that prolonged extrication (> 30 min) was associated with an increased need for emergency surgery (odds ratio 2.3 (1.2–4.6)).

Severe chest injuries are common in the trapped patients reported here. Chest injuries are often time sensitive and though they may be temporised by interventions such as supplemental oxygen, decompression of tension pneumothorax and analgesia, they are generally not amenable to definitive pre-hospital treatment. Delivering interventions is further hampered when a patient remains trapped in a vehicle, where oxygen may be contraindicated (due to ignition risk), technical procedures are difficult [17] and pauses for medical assessment and/or intervention further lengthen the time of extrication [18].

Those caring for patients who are trapped in cars should be aware of the frequency, severity and type of injuries which affect this patient group. FRS are often present at the scene prior to the arrival of an ambulance crew. Consideration should be given to how these personnel are trained and how their trauma skillset is relevant and proportional to this patient group.

### Limitations

Trapped patients are recorded on the TARN database as “patients that are involved in a vehicle collision and needed to be cut free”. Data entry personnel submitting data to TARN will rely on the “trapped” data box being completed on the ambulance service patient report form. It is not known how reliably this data is recorded on the patient report form and it cannot tell us if a patient was physically trapped or medically trapped*.* The inability of this dataset to determine between these groups of patients is a potential weakness of this study.

Approximately 88% of trapped patients are ‘medically trapped’, meaning they are unable to leave the vehicle due to pain, their injuries, or they are advised not to move in such circumstances [19]. This type of entrapment is also be termed ‘relative entrapment’.

Alternatively, patients may be ‘physically trapped’, which ordinarily refers to an event where the structure of the vehicle has changed by the application of external force preventing the patient from exiting the vehicle. This could be a simple issue, such as a door lock no longer working, or a more complex issue, for example a patient being pinned in the vehicle due to displacement of the dashboard. Where an impact is such that the internal structure of the car is displaced this is termed ‘intrusion’. An alternative term applied to patients physically trapped is ‘actual entrapment’ [20, 21]. Patients can also be physically trapped by external objects such as road furniture and there is an additional cohort of patients who are both physically and medically trapped.

Medically trapped patients would normally be extricated rapidly with minimal cutting of the vehicle whereas physically entrapped patients may require significant resource by the FRS before the patient can be extricated. Previous work in this area has identified that approximately 12% of patients are physically trapped, which is similar to the 11% we report here [19].

A further limitation of this study is that, by using TARN data, it does not include patients who were not eligible for TARN inclusion or patients that died at scene or in transit to hospital. Patients who die at the scene of an incident may have different injuries to those who survive to hospital admission e.g. airway obstruction or impact brain apnoea [22]. Review of coroners records have found the most common cause of death at scene was haemorrhage (35.7%), followed by neurotrauma (32.7%), and then combined haemorrhage and neurotrauma (31.6%) [23]. Inclusion of patients that died at scene would improve the robustness of these findings and give us further insight into and allow us to prioritise which interventions, training and extrication approaches should be prioritised to reduce the mortality associated with entrapment MVC.

## Conclusions

Trapped patients are more likely to die than those who are not trapped. The frequency of spinal cord injuries is low accounting for approximately 0.7% of all patients extricated. Patients who are trapped have a high rate of time-critical injuries requiring rapid intervention. Extrication takes time and when considering the frequency, type and severity of injuries reported here, the benefit of movement minimisation may be outweighed by the additional time taken. Improved extrication strategies should be developed which are evidence-based and allow for the expedient management of other life-threatening injuries.

## Data Availability

The datasets used and/or analysed during the current study are available from the corresponding author on reasonable request.
